# Antithrombotic therapy for secondary prevention of unprovoked venous thromboembolism: a systematic review and network meta-analysis of randomized controlled trials

**DOI:** 10.1080/07853890.2022.2026002

**Published:** 2022-01-13

**Authors:** Dandan Li, Yi Liu, Yao Song, Aiping Wen

**Affiliations:** Department of Pharmacy, Beijing Friendship Hospital, Capital Medical University, Beijing, China

**Keywords:** Secondary prevention, DOAC, unprovoked VTE, network meta-analysis

## Abstract

**Background:**

Extended antithrombotic treatment is recommended for secondary prevention of unprovoked venous thromboembolism (VTE), however, there is no consensus on which antithrombotic strategy is preferable.

**Aim:**

To compare the efficacy and safety of different antithrombotic strategies for secondary prevention unprovoked VTE.

**Methods:**

Cochrane Central Register of Controlled Trials, Embase, and MEDLINE were systematically searched from inception to 22 July 2020 for randomized controlled trials (RCTs) that compared the efficacy and/or safety of extended antithrombotic strategies including aspirin, warfarin and direct oral anticoagulants (DOACs) for secondary prevention of unprovoked VTE. The primary outcome was risk of major bleeding and the secondary outcomes were risks of recurrent VTE and all-cause death. Odds ratios (ORs) and 95% confidence intervals (CIs) were estimated using pairwise and network meta-analysis with random effect. Possible ranking of extended antithrombotic strategies was plotted using the surface under the cumulative ranking curve and mean ranks.

**Results:**

Seventeen RCTs met the inclusion criteria, and meta-analysis results showed that warfarin was associated with significantly higher risk of major bleeding than placebo/observation (OR 2.71, 95% CI 1.32–5.55) or apixaban (OR 10.65, 95% CI 1.06–107.13). Apixaban and low-apixaban were the top two strategies according to the ranking of major bleeding. Warfarin (OR 0.25, 95%CI 0.13–0.49), rivaroxaban (OR 0.18, 95%CI 0.03–0.90), apixaban (OR 0.18, 95%CI 0.04–0.85) and low-apixaban (OR 0.18, 95%CI 0.04–0.82) were related to significantly lower risk than placebo/observation; edoxaban was non-inferior to warfarin on the risk of recurrent VTE. Furthermore, apixaban was linked with significantly lower risk of all-cause death than placebo/observation (OR 0.29, 95% CI 0.09–0.88).

**Conclusion:**

Apixaban showed superiority to other antithrombotic strategies on major bleeding and all-cause death for secondary prevention of unprovoked VTE. Further studies are warranted owing to the limited number of studies and positive cases.

**Key messages**
1. All antithrombotic strategies including warfarin, DOACs and aspirin were superior to placebo/observation on recurrent VTE for secondary prevention of unprovoked VTE.
2. Apixaban demonstrated lower risk of major bleeding than warfarin, and lower risk of all-cause death than placebo/observation.
3. Further research about the efficacy and safety of antithrombotic treatments for secondary prevention of unprovoked VTE is warranted.

Venous thromboembolism (VTE), clinically presenting as deep vein thrombosis (DVT) or pulmonary embolism (PE), is associated with significant mortality, morbidity, and economic burden globally [1–3]. Patients with unprovoked (also termed as “idiopathic”) VTE, which refers to cases without presence of risk factors, such as surgery, trauma or immobilization [4], are faced with much higher or even doubled risk of VTE recurrence than the provoked cases once anticoagulation is stopped [5,6]. It is estimated that the risk of recurrent VTE is 10% in the first year, 16% at 2 years, 25% at 5 years, and 36% at 10 years for patients with unprovoked VTE who completed at least 3 months of anticoagulant treatment [7].

The updated guideline from the American Society of Haematology (ASH) conditionally recommends continuing indefinite antithrombotic treatment for unprovoked VTE patients with lower risk of bleeding [8]. However, antithrombotics are related with increased risk of bleedings, including gastrointestinal bleeding [4], intracranial haemorrhage [9], etc. The estimated case-fatality rate for major haemorrhage can be as high as 11.3% [10]. Thus, it is important to understand the benefit and bleeding risk of different antithrombotic strategies during the secondary prevention of unprovoked VTE.

Though several meta-analyses have been published, they are limited by inclusion of both provoked and unprovoked patients, or pooling all direct oral anticoagulants (DOACs) in one treatment arm, possibly introducing bias and unable to demonstrating the difference among DOACs [11–14]. In this study, we aimed to assess the efficacy and safety of extended antithrombotic strategies including aspirin, warfarin and DOACs for patients with unprovoked VTE with pairwise and network meta-analysis.

## Methods

This systematic review was prepared according to the Preferred Reporting Items for Systematic Reviews and Meta-Analysis (PRISMA) [15] as well as the PRISMA extension statement for network meta-analysis [16].

### Search strategy and selection criteria

We searched Embase (1947 to 22 July 2020) and MEDLINE (1946 to 22 July 2020) using the OVID interface, and the Cochrane Central Register of Controlled Trials (inception to July 2020), with restriction to English language. Search terms included “thrombosis,” “deep vein thrombosis,” “pulmonary embolism,” “anticoagulant,” etc., details are shown in Table S1.

The inclusion criteria of randomized controlled trials (RCTs) were as follows: (1) the percentage of patients with unprovoked VTE need to be more than 50%; (2) evaluated the efficacy and safety of secondary prevention of antithrombotics after 3–6 months of primary treatment or the total antithrombotic duration was longer than 3 months. The antithrombotic strategies included aspirin, warfarin and DOACs. We excluded studies that involved VTEs treated with anticoagulant agents that are omitted from the market (e.g. ximelagatran), and studies that evaluated the anticoagulation of acute phase of VTE. References of included studies and narrative reviews were read for additional potential studies.

The predefined primary outcomes were risk of major bleeding, defined as overt bleedings and associated with a decrease in haemoglobin of 2 g per decilitre or more or required a transfusion of 2 or more units of blood, occurred in a critical site, or contributed to death. The secondary outcomes were risks of recurrent VTE and all-cause death of different antithrombotic strategies.

### Data extraction and quality assessment

Two reviewers (D. L. and Y. L.) independently screened titles and abstracts of the retrieved studies to exclude those did not explore questions of interest, and then independently screened full texts of the remaining studies to identify those met all of the inclusion criteria. For each included trial, two reviewers independently extracted the characteristics of the included studies and patients, as well as outcome measures as predefined. Discrepancies were resolved by discussing them with the third reviewer (A. W.).

Intention to treat analysis (ITT) results were extracted wherever possible. If ITT data was not available, we used data that the author reported. Unless unavailable, we extracted data reported at the end of treatment. If one article published two or more subgroups with independent randomization approach, they would be considered separately.

As the international normalized ratio (INR) of warfarin was routinely adjusted to be 2.0–3.0 for patients with VTE, the target INR of 1.5–2.0 was deemed as low intensity, and abbreviated as “low-warfarin.” Similarly, the dose of 5 mg/d (2.5 mg bid) of apixaban were abbreviated as “low-apixaban.” Participants discontinuing antithrombotic therapy were classified as “placebo/observation” group: “placebo” refers to those received placebo, while “observation” refers those did not take any antithrombotics or placebo.

The quality of the included studies was assessed using the Cochrane Collaboration’s tool for assessing the risk of bias [17]. Two reviewers assessed the risk of bias independently and in duplicate; any disagreements were resolved in consultation with the supervisor.

### Data synthesis and statistical analysis

Bayesian network meta-analyses and direct frequentist pairwise meta-analyses were conducted for all outcomes with STATA 13.1 (StataCorp, College Station, TX). As only dichotomous outcomes were involved, odds ratios (OR) and 95% confidence interval (CI) were calculated based on random-effect model. The heterogeneity was evaluated using the *I^2^* statistic (low: < 25%, moderate: 25–75%, high: >75%). Meta-regression was conducted to test effects of covariates on intended outcomes. Publication bias was assessed by funnel plot symmetry with Egger's test. With the “network” command in STATA [18], we assessed the global inconsistency with the “design-by-treatment” model and loop-specific inconsistency within each loop. To rank the superiority of interventions, we also plotted the surface under the cumulative ranking curves (SUCRAs) and mean ranks [19]. As RCTs involving more than 50% of unprovoked VTE were included in this study, we conducted a sensitivity analysis by pooling RCTs of 100% unprovoked VTE patients. A two-sided *p* < .05 was considered significant.

## Results

### Characteristics of included studies and quality assessment

**Figure 1.**
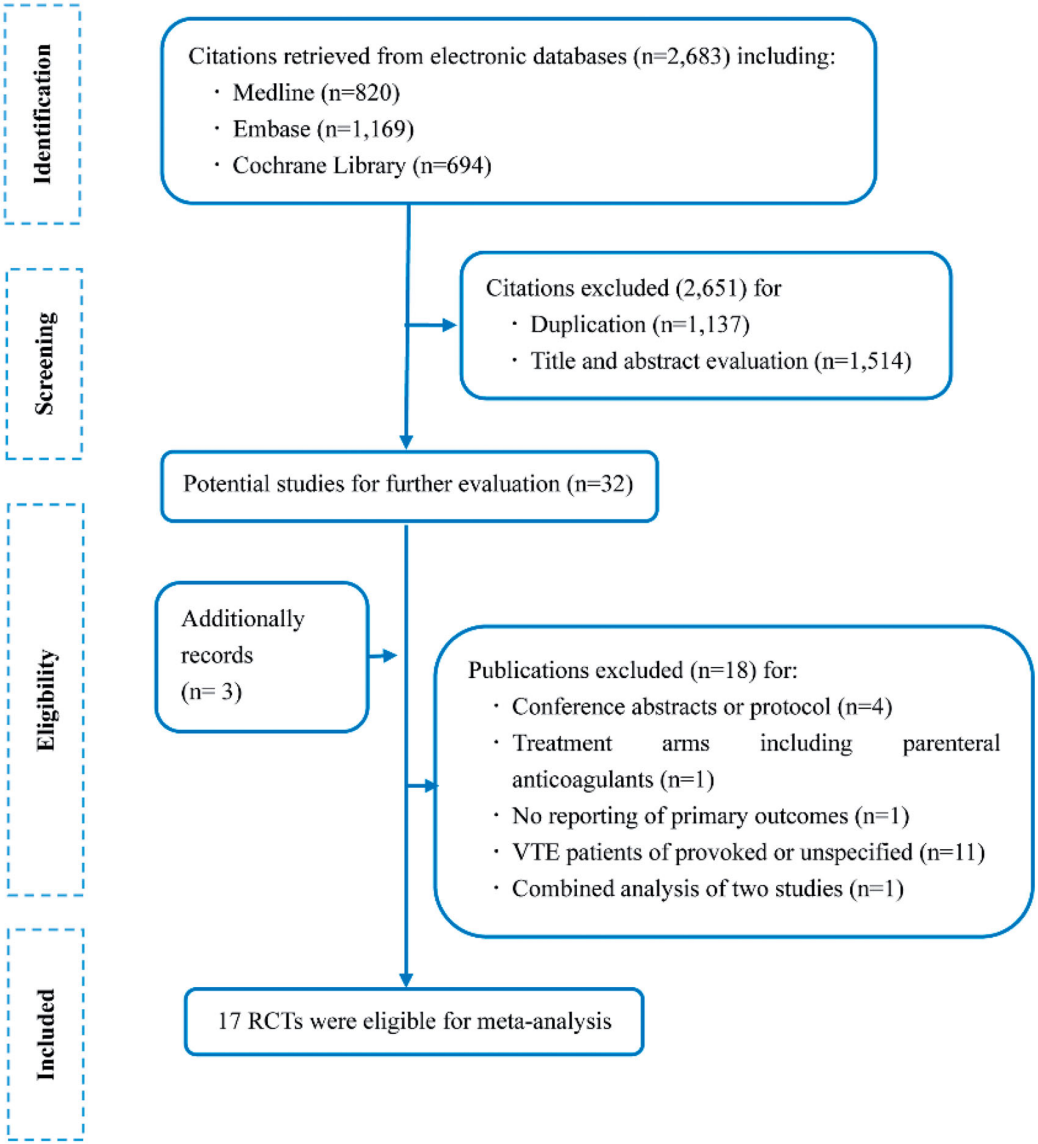
Flow diagram of study selection.

About 2,683 citations were identified by electronic search and 32 potentially eligible articles were retrieved for full text screen (Figure 1). Finally, we included 14 trials from the database search, and 3 from hand-searching of other review articles. Table 1 summarized the baseline characteristics of included studies. Thirteen studies recruited patients with unprovoked VTE only, whereas the proportion of unprovoked VTE was about 72% (range 66–91%) in four studies. The mean age of these studies varied from 41.5 to 69.7 years old. The percentage of male ranged from 45.0% to 75.7%. Majority of patients are the white race except that Farraj [20] included participants from Jordon. The duration of secondary prevention ranged from 0 to 48 m.

**Table 1.** Baseline characteristic of the included studies.

Author (year)	*N*	First episode	Unprovoked VTE (%)	VTE categories	Age (mean, year)	Male (%)	Race (primary)	Interventions	Duration (month)
Agnelli 2001 [21]	267	Yes	100	DVT	67.2	57.8	White	Observation vs Warfarin	9
Agnelli 2003^a,b^ [22]	181	Yes	100	PE	67.0	75.7	White	Observation vs Warfarin	9
Agnelli 2013 [23]	2482	No	91	VTE	56.7	57.4	White	Placebo vs low-apixaban vs Apixaban	12
Bauersachs 2010 [24]	1196	No	74	VTE	58.3	58.0	White	Placebo vs Rivaroxaban	6–12
Becattini 2012 [25]	402	Yes	100	VTE	61	64	White	Placebo vs Aspirin	24
Bradbury 2020 [26]	273	Yes	100	VTE	62.7	67.4	White	Observation vs Warfarin	24
Brighton 2012 [27]	822	Yes	100	VTE	54	54	White	Placebo vs Aspirin	48
Buller 2013 [28]	8240	No	66	VTE	55.8	57.2	White	Edoxaban vs Warfarin	3–12^c^
Couturaud 2015 [29]	371	Yes	100	PE	58.5	48.8	White	Placebo vs Warfarin	18
Couturaud 2019 [30]	104	Yes	100	DVT	60.3	67.3	White	Placebo vs Warfarin	18
Eischer 2009 [31]	34	Yes	100	VTE	53.5	53.0	White	Observation vs Warfarin	24
Farraj 2004 [20]	64	Yes	100	VTE	41.5	59.4	Asian	Observation vs Warfarin	18
Kearon 1999 [32]	162	Yes	100	VTE	59	60.5	White	Placebo vs Warfarin	24
Kearon 2003 [33]	738	No	100	VTE	57.0	45.0	White	Low-warfarin vs Warfarin	28.8
Palareti 2006^a^ [34]	223	Yes	100	VTE	69.7	47.1	White	Observation vs Warfarin	18
Ridker 2003 [35]	508	NA	100	VTE	53^d^	47.2	White	Placebo vs low-warfarin	25.2
Siragusa 2008^a^ [36]	180	Yes	77	DVT	57.1	52.8	White	Observation vs Warfarin	9

VTE: venous thromboembolism including deep-vein thrombosis and pulmonary embolism; DVT: deep-vein thrombosis; PE: pulmonary embolism.

^a^Studies that only subgroup were included.

^b^Studies that reported events of interested after the extended follow-up after cession of treatment.

^c^61.5% of included patients were treated for 12 months.

^d^Median age. Low-warfarin: warfarin with international ratio of 1.5–2.0.

The risk of bias assessment was performed for each RCT and summarized in Figure S1. Most of the studies were in the lowest categories of risk of bias for random sequence generation (13/17, 82.4%), blinding of participants and personnel (10/17, 58.8%), blinding of outcome assessment (15/17, 88.2%), incomplete outcome data (15/17, 88.2%), selective reporting (10/17, 58.8%) and other bias (10/17, 58.8%). Allocation concealment was unclear in 52.9% (9/17) of included RCTs.

### Major bleeding of different antithrombotic strategies

**Figure 2.**
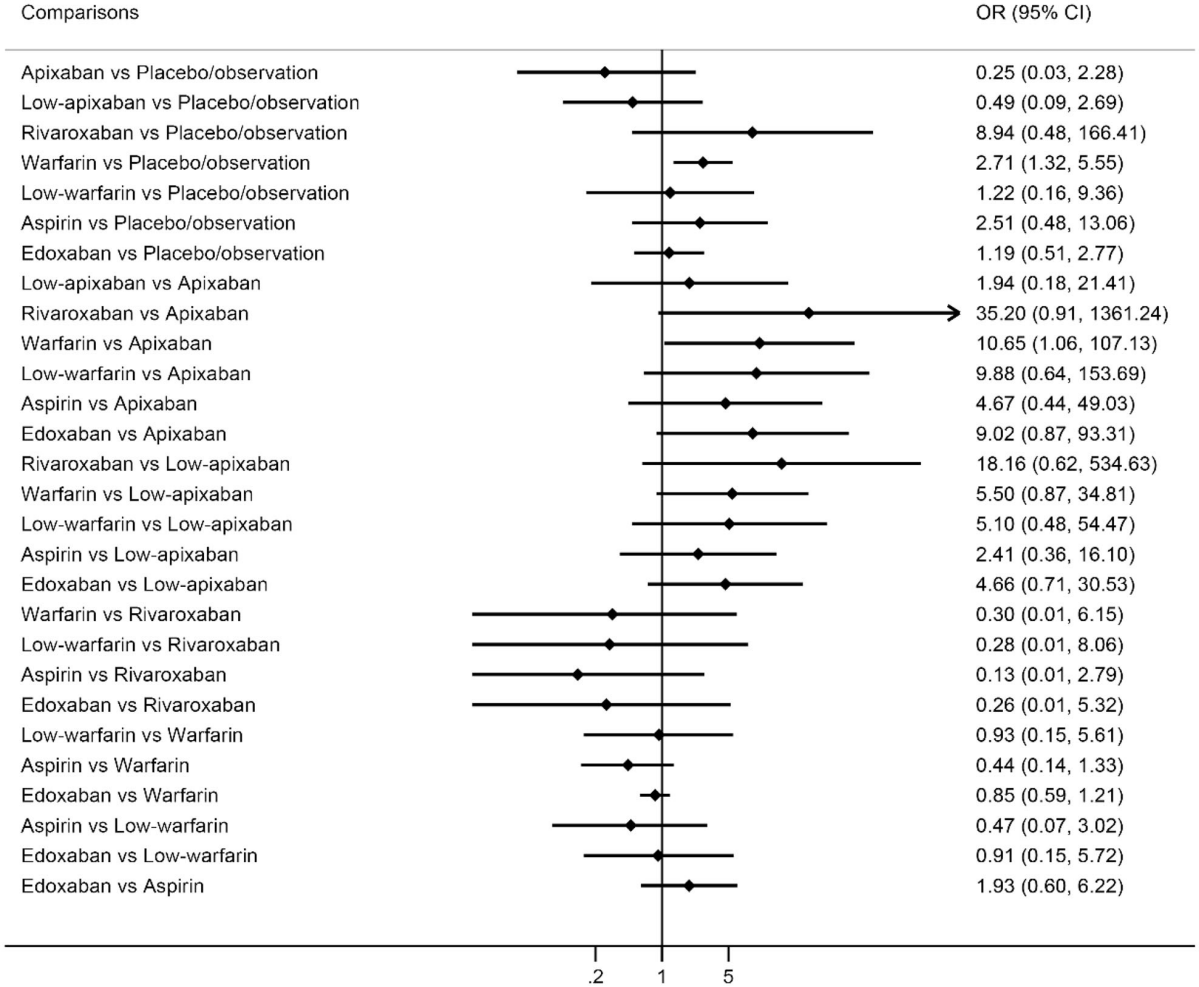
Network meta-analysis results of major bleeding of different antithrombotic strategies for unprovoked venous thromboembolism. OR: odds ratio; CI: confidence interval.

All included studies comprising 16,247 patients reported the risk of major bleeding (Figure S2). Network meta-analysis indicated that warfarin was associated with significantly higher risk of major bleeding than placebo/observation (OR 2.71, 95% CI 1.32–5.55) or apixaban (OR 10.65, 95% CI 1.06–107.13). There was no significant difference between other comparisons (Figure 2). The ranking of different antithrombotic strategies based on SUCRAs and mean ranks showed that apixaban and low-apixaban were associated with lower risk of major bleeding than other antithrombotic strategies or even placebo/observation (Table S2).

Global (Table S3) and loop-specific inconsistency were not detected in any comparisons. We didn’t find the modification effect of race, ag, gender, episodes (first or not), unprovoked VTE percentage, VTE categories (PE, DVT or both) or antithrombotic duration on major bleeding from meta-regression (Table S4). Publication bias by Egger’s test was significant (*p* = .005).

Pairwise meta-analysis results are illustrated in Table S5. Similar with the network meta-analysis, warfarin was associated with significantly higher risk of major bleeding than placebo/observation (OR 2.79, 95% CI 1.34–5.80).

### Recurrent VTE and all-cause death of different antithrombotic strategies

Network meta-analysis showed that warfarin (OR 0.25, 95%CI 0.13–0.49), rivaroxaban (OR 0.18, 95%CI 0.03–0.90), apixaban (OR 0.18, 95%CI 0.04–0.85) and low-apixaban (OR 0.18, 95%CI 0.04–0.82) can all significantly reduce the risk of recurrent VTE than placebo/observation (Table S6A). Furthermore, apixaban was associated with significantly lower risk of all-cause death than placebo/observation (OR 0.29, 95% CI 0.09–0.88) (Table S6B). No significant differences were found between comparisons on risks of recurrent VTE and all-cause death.

According to the rankings based on SUCRAs and mean ranks (Table S2), DOACs including apixaban, low-apixaban, rivaroxaban and edoxaban are superior to warfarin on recurrent VTE; and all the antithrombotic strategies demonstrated better efficacy on recurrent VTE and all-cause death than placebo/observation.

Global (Table S3) and loop-specific inconsistency was not detected in any comparisons. Publication bias for recurrent VTE and all-cause death were 0.019 and 0.211, respectively. According to meta-regression results, no modification effect of race, age, gender, episodes (first or not), unprovoked VTE percentage, VTE categories (PE, DVT or both) or antithrombotic duration was found for all comparisons (Table S4).

Similar with the network meta-analysis, the pairwise meta-analysis results showed that warfarin (OR 0.24, 95% CI 0.12–0.49), rivaroxaban (OR 0.18, 95% CI 0.08–0.38), apixaban (OR 0.18, 95% CI 0.10–0.32) and low-apixaban (OR 0.18, 95% CI 0.10–0.31) were associated with significantly lower risk of recurrent VTE than placebo/observation. Furthermore, aspirin (OR 0.68, 95% CI 0.50–0.92) and low-warfarin (OR 0.34, 95% CI 0.18–0.64) were associated with significantly lower risk of recurrent VTE than placebo/observation, low-warfarin was linked with significantly higher risk of recurrent VTE compared with warfarin (OR 2.74, 95% CI 1.06–7.09).

During the sensitivity analysis of studies that including 100% unprovoked VTE patients, no DOACs were involved (Figure S3). Both warfarin (OR 2.69, 95% CI 1.33–5.42) and low-warfarin (OR 2.89, 95% CI 1.08–7.72) were associated with significant higher risk of major bleeding than placebo/observation. Warfarin was linked with significantly lower risk of recurrent VTE than aspirin (OR 0.31, 95% CI 0.10–0.96) and placebo/observation (OR 0.21, 95% CI 0.11–0.38).

## Discussion

### Major findings and clinical implications

Our study indicated that all anticoagulant strategies including warfarin and DOACs were linked with significant lower risk of recurrent VTE than placebo/observation. Apixaban was associated with significantly lower risk of major bleeding than warfarin and lower risk of all-cause death than placebo/observation.

Current guidelines recommended extended or indefinite antithrombotics for secondary prevention of unprovoked VTE in the updated guidelines [8,37]. However, there are still controversaries considering the choice of antithrombotic strategies: the European Society of Cardiology recommended DOACs as the first-line anticoagulants [37,38], while the American College of Chest Physicians and ASH guideline recommended continuing indefinite anticoagulation with the same drug administered during the first months without specifying particular one [8,39].

Our study indicated that all anticoagulant treatments could reduce the risk of recurrent VTE, which is consistent with previous studies [40,41]. Thus, any anticoagulant drugs can be chosen once available. For patients with poor adhere or polypharmacy, DOACs may be a better choice than warfarin for its need to frequently monitor of INR [42] and the potential drug–drug or drug–food interactions [43].

According to the rankings of major bleeding, apixaban and low-apixaban showed better safety than rivaroxaban and edoxaban. This was verified by real-world evidence: Jin MC identified 225,559 VTE patients receiving anticoagulation from the Optum Clinformatics Data Mart (2003–2019) of the US, and found that apixaban was associated with significantly reduced non-intracranial haemorrhage and recurrent VTE risk compared with rivaroxaban [44]. In addition, we found apixaban was associated with significantly lower risk of all-cause death than placebo/observation. Thus, for patients with higher risk of bleeding, apixaban might be a better choice. Even though no predictive scores were currently recommended, the risk factors that associated with increased risk of major bleeding included older age, female sex, abnormal creatinine levels, anaemia, PE diagnosis at baseline, etc. [44,45]

AMPLIFY-EXT study evaluated the effect of low-apixaban, apixaban versus placebo, and demonstrated that both dose of apixaban were superior to placebo on recurrent VTE [23]. The ESC guidelines [38] also recommended using low dose of apixaban (2.5 mg b.i.d.) and rivaroxaban (10 mg o.d.) for extended oral anticoagulation of PE. Thus, for patients cannot tolerate the standard dose of apixaban or rivaroxaban, lower dose might be an alternative.

On the contrary, the updated ACH guideline strongly recommended that for patients with DVT and/or PE who will use warfarin therapy, a standard intensity of warfarin was over a lower intensity [8]. Even though we did not find significant difference between warfarin and low dose of warfarin, Kearon C [33] found that compared with the standard intensity, the lower intensity of warfarin was associated with higher risk of recurrent VTE without significant benefit on major bleeding.

### Comparison with other studies

Many meta-analyses have compared the efficacy and safety of different antithrombotic strategies for secondary prevention of VTE patients including the provoked and unprovoked: Kakkos [11] assessed the DOACs versus placebo, and found that DOACs can reduced recurrent VTE and all-cause mortality at the expense of higher risk of clinically relevant non-major bleeding. Alotaibi [12] compared different DOACs, and found no significant differences in risk for recurrent VTE, major bleeding, or all-cause mortality. Using network meta-analysis method, Sobieraj [13] found that oral anticoagulants (including apixaban, dabigatran, rivaroxaban, and warfarin) and idraparinux were superior to placebo on recurrent VTE, while Rollins [14] found that no differences among oral anticoagulants and placebo on the composite end point of VTE or death, nonfatal PE, or DVT. However, apixaban demonstrated a more favourable safety profile compared to other therapies in both studies [13,14].

As our understanding of VTE increased, more attentions were needed for unprovoked VTE because of its higher risk of recurrence [7]. Holley [46] and Bova [47] have compared the prolonged versus shorter antithrombotic durations for unprovoked VTE, but they did not compare the differences of different anticoagulant strategies. Sindet-Pedersen [48] aimed to examine the safety and efficacy of different anticoagulant strategies versus placebo. Marik [41] furtherly added aspirin into their study protocol. However, as there were limited head-to-head studies, authors were not able to compare the effect of DOACs versus warfarin with pairwise meta-analysis method. Mai [49] conducted a network meta-analysis to evaluate the pharmacologic therapies for extended anticoagulation of unprovoked VTE. However, studies with less than 50% or unknown percentage of unprovoked VTE patients were also included. In this study, we have comprehensively searched the newly published RCTs, and rigorously adopted standard of >50% because it is widely used in other analyses [50]. In addition, we performed sensitivity analysis to strengthen the robustness of the results, and meta-regression to explore possible factors associated with intended outcomes.

## Strengths and limitations

To our knowledge, this is the largest and most comprehensive systematic review and network meta-analysis of RCTs to explore the efficacy and safety of antithrombotic strategies for secondary prevention of unprovoked VTE. Of course, we acknowledge the following limitations of this study. First of all, the number of included studies and positive cases were limited, especially in each intervention arms. For example, among included studies, only AMPLIFY-EXT study evaluated the effect of apixaban on major bleeding. There were only one positive case for apixaban and two positive cases for low-apixaban, which may introduce bias. However, we have followed the inclusion criteria strictly. RE-MEDY and RE-SONATE studies have assessed effects of dabigatran versus warfarin or placebo during the extended anticoagulation of VTE [51], we did not include these RCTs because either the publication [51] or the registered website [52] reported the actual percentage of unprovoked patients. EINSTEIN CHOICE [53] study, which evaluated the effects of standard as well as lower dose of rivaroxaban versus aspirin, was also excluded because more than half of the included VTE patients were provoked [53]. Second, the baseline characteristics of included participants are different among included studies. For example, Eischer [31] included VTE patients with high factor VIII (FVIII) (>230 IU/dL); we had only included patients with elevated d-dimer in the PROLONG study [34]. In addition, the percentage of unprovoked VTE ranged from 66% to 100%, and some studies have included only patients with first episode of VTE while others didn’t. We aimed to conduct a sensitivity analysis of studies including 100% unprovoked patients, however, there were no DOACs involved. Thirdly, the lengths of treatment varied across studies. As the risk of recurrent VTE varied in different years after initial treatment, collecting the data as authors reported may introduce bias though meta-regression found no modification effect of antithrombotic duration.

## Conclusions

This study supported indefinite antithrombotic treatment because of their superiority on recurrent VTE to placebo/observation. When facing patients with relatively high risk of bleeding, apixaban or low dose of apixaban might be good choices as their advantages on major bleeding. Further studies are warranted owing to the limited number of studies and positive cases.

## Supplementary Material

Supplemental Material

## Data Availability

The authors confirm that the data supporting the findings of this study are available within the article and its Supplementary materials. The raw data of this study are available from the corresponding author (A. W.) upon reasonable request.

## References

[1] Raskob GE, Angchaisuksiri P, Blanco AN, et al. Thrombosis: a major contributor to global disease burden. Arterioscler Thromb Vasc Biol. 2014;34:2363–2371. 25304324 10.1161/ATVBAHA.114.304488

[2] Naess IA, Christiansen SC, Romundstad P, et al. Incidence and mortality of venous thrombosis: a population-based study. J Thromb Haemost. 2007;5:692–699. 17367492 10.1111/j.1538-7836.2007.02450.x

[3] Beckman MG, Hooper WC, Critchley SE, et al. Venous thromboembolism: a public health concern. Am J Prev Med. 2010;38:S495–S501. 20331949 10.1016/j.amepre.2009.12.017

[4] Bouget J, Viglino D, Yvetot Q, et al. Major gastrointestinal bleeding and antithrombotics: characteristics and management. World J Gastroenterol. 2020;26:5463–5473. 33024397 10.3748/wjg.v26.i36.5463 PMC7520611

[5] Prandoni P, Noventa F, Ghirarduzzi A, et al. The risk of recurrent venous thromboembolism after discontinuing anticoagulation in patients with acute proximal deep vein thrombosis or pulmonary embolism. A prospective cohort study in 1,626 patients. Haematologica. 2007;92:199–205. 17296569 10.3324/haematol.10516

[6] Boutitie F, Pinede L, Schulman S, et al. Influence of preceding length of anticoagulant treatment and initial presentation of venous thromboembolism on risk of recurrence after stopping treatment: analysis of individual participants' data from seven trials. BMJ. 2011;342:d3036. 21610040 10.1136/bmj.d3036 PMC3100759

[7] Khan F, Rahman A, Carrier M, et al. Long term risk of symptomatic recurrent venous thromboembolism after discontinuation of anticoagulant treatment for first unprovoked venous thromboembolism event: systematic review and meta-analysis. BMJ. 2019;366:l4363. 31340984 10.1136/bmj.l4363 PMC6651066

[8] Ortel TL, Neumann I, Ageno W, et al. American Society of Hematology 2020 guidelines for management of venous thromboembolism: treatment of deep vein thrombosis and pulmonary embolism. Blood Adv. 2020;4:4693–4738. 33007077 10.1182/bloodadvances.2020001830 PMC7556153

[9] Macdonald RL. Management of intracranial hemorrhage in the anticoagulated patient. Neurosurg Clin N Am. 2018;29:605–613. 30223973 10.1016/j.nec.2018.06.013

[10] Carrier M, Le Gal G, Wells PS, et al. Systematic review: case-fatality rates of recurrent venous thromboembolism and major bleeding events among patients treated for venous thromboembolism. Ann Intern Med. 2010;152:578–589. 20439576 10.7326/0003-4819-152-9-201005040-00008

[11] Kakkos SK, Kirkilesis GI, Tsolakis IA. Editor's choice – efficacy and safety of the new oral anticoagulants dabigatran, rivaroxaban, apixaban, and edoxaban in the treatment and secondary prevention of venous thromboembolism: a systematic review and meta-analysis of phase III trials. Eur J Vasc Endovasc Surg. 2014;48:565–575. 24951377 10.1016/j.ejvs.2014.05.001

[12] Alotaibi G, Alsaleh K, Wu C, et al. Dabigatran, rivaroxaban and apixaban for extended venous thromboembolism treatment: network meta-analysis. Int Angiol. 2014;33:301–308. 25056161

[13] Sobieraj DM, Coleman CI, Pasupuleti V, et al. Comparative efficacy and safety of anticoagulants and aspirin for extended treatment of venous thromboembolism: a network meta-analysis. Thromb Res. 2015;135:888–896. 25795564 10.1016/j.thromres.2015.02.032

[14] Rollins BM, Silva MA, Donovan JL, et al. Evaluation of oral anticoagulants for the extended treatment of venous thromboembolism using a mixed-treatment comparison, meta-analytic approach. Clin Ther. 2014;36:1454–1464 e1453. 25092394 10.1016/j.clinthera.2014.06.033

[15] Moher D, Liberati A, Tetzlaff J, et al. Preferred reporting items for systematic reviews and meta-analyses: the PRISMA statement. Ann Intern Med. 2009;151(4):264–269. W264. 19622511 10.7326/0003-4819-151-4-200908180-00135

[16] Hutton B, Salanti G, Caldwell DM, et al. The PRISMA extension statement for reporting of systematic reviews incorporating network meta-analyses of health care interventions: checklist and explanations. Ann Intern Med. 2015;162:777–784. 26030634 10.7326/M14-2385

[17] Higgins J, Sterne AD. Chapter 8: Assessing risk of bias in included studies. In: Higgins J, Green S, editors. Cochrane handbook for systematic reviews of interventions, 5.1.0 ed. London: The Cochrane Collaboration. Available from: https://handbook-5-1.cochrane.org/2011

[18] White IR. Network meta-analysis. Stata J. 2015;15(4):951–985.

[19] Salanti G, Ades AE, Ioannidis JP. Graphical methods and numerical summaries for presenting results from multiple-treatment meta-analysis: an overview and tutorial. J Clin Epidemiol. 2011;64:163–171. 20688472 10.1016/j.jclinepi.2010.03.016

[20] Farraj RS. Anticoagulation period in idiopathic venous thromboembolism. How long is enough? Saudi Med J. 2004;25:848–851. 15235686

[21] Agnelli G, Prandoni P, Santamaria MG, et al. Three months versus one year of oral anticoagulant therapy for idiopathic deep venous thrombosis. N Engl J Med. 2001;345:165–169. 11463010 10.1056/NEJM200107193450302

[22] Agnelli G, Prandoni P, Becattini C, et al. Extended oral anticoagulant therapy after a first episode of pulmonary embolism. Ann Intern Med. 2003;139:19–25. 12834314 10.7326/0003-4819-139-1-200307010-00008

[23] Agnelli G, Buller HR, Cohen A, et al. Apixaban for extended treatment of venous thromboembolism. N Engl J Med. 2013;368(8):699–708. 23216615 10.1056/NEJMoa1207541

[24] Bauersachs R, Berkowitz SD, Brenner B, et al. Oral rivaroxaban for symptomatic venous thromboembolism. N Engl J Med. 2010;363:2499–2510. 21128814 10.1056/NEJMoa1007903

[25] Becattini C, Agnelli G, Schenone A, et al. Aspirin for preventing the recurrence of venous thromboembolism. N Engl J Med. 2012;366:1959–1967. 22621626 10.1056/NEJMoa1114238

[26] Bradbury C, Fletcher K, Sun Y, et al. A randomised controlled trial of extended anticoagulation treatment versus standard treatment for the prevention of recurrent venous thromboembolism (VTE) and post-thrombotic syndrome in patients being treated for a first episode of unprovoked VTE (the ExACT study. Br J Haematol. 2020;188(6):962–975. ). 31713863 10.1111/bjh.16275

[27] Brighton TA, Eikelboom JW, Mann K, et al. Other source: low-dose aspirin for preventing recurrent venous thromboembolism. N Engl J Med. 2012;367:1979–1987. 23121403 10.1056/NEJMoa1210384

[28] Buller HR, Decousus H, Grosso MA, et al. Edoxaban versus warfarin for the treatment of symptomatic venous thromboembolism. N Engl J Med. 2013;369:1406–1415. 23991658 10.1056/NEJMoa1306638

[29] Couturaud F, Sanchez O, Pernod G, et al. Six months vs extended oral anticoagulation after a first episode of pulmonary embolism: the PADIS-PE randomized clinical trial. JAMA. 2015;314:31–40. 26151264 10.1001/jama.2015.7046

[30] Couturaud F, Pernod G, Presles E, for the “PADIS-DVT” Investigators, et al. Six months versus two years of oral anticoagulation after a first episode of unprovoked deep-vein thrombosis. The PADIS-DVT randomized clinical trial. Haematologica. 2019;104(7):1493–1501. 30606789 10.3324/haematol.2018.210971 PMC6601089

[31] Eischer L, Gartner V, Schulman S, et al. 6 Versus 30 months anticoagulation for recurrent venous thrombosis in patients with high factor VIII. Ann Hematol. 2009;88:485–490. 18931845 10.1007/s00277-008-0626-1

[32] Kearon C, Gent M, Hirsh J, et al. A comparison of three months of anticoagulation with extended anticoagulation for a first episode of idiopathic venous thromboembolism. N Engl J Med. 1999;340(12):901–907. 10089183 10.1056/NEJM199903253401201

[33] Kearon C, Ginsberg JS, Kovacs MJ, et al. Comparison of low-intensity warfarin therapy with conventional-intensity warfarin therapy for long-term prevention of recurrent venous thromboembolism. N Engl J Med. 2003;349:631–639. 12917299 10.1056/NEJMoa035422

[34] Palareti G, Cosmi B, Legnani C, et al. D-dimer testing to determine the duration of anticoagulation therapy. N Engl J Med. 2006;355:1780–1789. 17065639 10.1056/NEJMoa054444

[35] Ridker PM, Goldhaber SZ, Danielson E, et al. Long-term, low-intensity warfarin therapy for the prevention of recurrent venous thromboembolism. N Engl J Med. 2003;348(15):1425–1434. 12601075 10.1056/NEJMoa035029

[36] Siragusa S, Malato A, Anastasio R, et al. Residual vein thrombosis to establish duration of anticoagulation after a first episode of deep vein thrombosis: the duration of anticoagulation based on compression ultrasonography (DACUS) study. Blood. 2008;112:511–515. 18497320 10.1182/blood-2008-01-131656

[37] Mazzolai L, Aboyans V, Ageno W, et al. Diagnosis and management of acute deep vein thrombosis: a joint consensus document from the European Society of Cardiology Working groups of aorta and peripheral vascular diseases and pulmonary circulation and right ventricular function. Eur Heart J. 2018;39:4208–4218. 28329262 10.1093/eurheartj/ehx003

[38] Konstantinides SV, Meyer G, Becattini C, et al. 2019 ESC guidelines for the diagnosis and management of acute pulmonary embolism developed in collaboration with the European Respiratory Society (ERS). Eur Heart J. 2020;41:543–603. 31504429 10.1093/eurheartj/ehz405

[39] Kearon C, Akl EA, Ornelas J, et al. Antithrombotic therapy for VTE disease: CHEST guideline and expert panel report. Chest. 2016;149:315–352. 26867832 10.1016/j.chest.2015.11.026

[40] Middeldorp S, Prins MH, Hutten BA. Duration of treatment with vitamin K antagonists in symptomatic venous thromboembolism. Cochrane Database Syst Rev. 2014;CD001367. 25092359 10.1002/14651858.CD001367.pub3 PMC7074008

[41] Marik PE, Cavallazzi R. Extended anticoagulant and aspirin treatment for the secondary prevention of thromboembolic disease: a systematic review and meta-analysis. PLoS One. 2015;10(11):e0143252. 26587983 10.1371/journal.pone.0143252 PMC4654552

[42] Jacobs LG. Warfarin pharmacology, clinical management, and evaluation of hemorrhagic risk for the elderly. Cardiol Clin. 2008;26:157–167. 18406992 10.1016/j.ccl.2007.12.010

[43] Di Minno A, Frigerio B, Spadarella G, et al. Old and new oral anticoagulants: food, herbal medicines and drug interactions. Blood Rev. 2017;31:193–203. 28196633 10.1016/j.blre.2017.02.001

[44] Jin MC, Sussman ES, Feng AY, et al. Hemorrhage risk of direct oral anticoagulants in real-world venous thromboembolism patients. Thromb Res. 2021;204:126–133. 34198049 10.1016/j.thromres.2021.06.015

[45] Ruiz-Gimenez N, Suarez C, Gonzalez R, et al. Predictive variables for major bleeding events in patients presenting with documented acute venous thromboembolism. Findings from the RIETE Registry. Thromb Haemost. 2008;100:26–31. 18612534 10.1160/TH08-03-0193

[46] Holley AB, King CS, Jackson JL, et al. Different finite durations of anticoagulation and outcomes following idiopathic venous thromboembolism: a meta-analysis. Thrombosis. 2010;2010:540386. 22084660 10.1155/2010/540386 PMC3211079

[47] Bova C, Bianco A, Mascaro V, et al. Extended anticoagulation and mortality in venous thromboembolism. A meta-analysis of six randomized trials. Thromb Res. 2016;139:22–28. 26916292 10.1016/j.thromres.2016.01.005

[48] Sindet-Pedersen C, Pallisgaard JL, Olesen JB, et al. Safety and efficacy of direct oral anticoagulants compared to warfarin for extended treatment of venous thromboembolism – a systematic review and meta-analysis. Thromb Res. 2015;136:732–738. 26277682 10.1016/j.thromres.2015.07.022

[49] Mai V, Bertoletti L, Cucherat M, et al. Extended anticoagulation for the secondary prevention of venous thromboembolic events: an updated network meta-analysis. PLoS One. 2019;14:e0214134. 30933993 10.1371/journal.pone.0214134 PMC6443183

[50] Pereira NL, Rihal C, Lennon R, et al. Effect of CYP2C19 genotype on ischemic outcomes during oral P2Y12 inhibitor therapy: a meta-analysis. JACC Cardiovasc Interv. 2021;14(7):739–750. 33744207 10.1016/j.jcin.2021.01.024 PMC9853943

[51] Schulman S, Kearon C, Kakkar AK, et al. Extended use of dabigatran, warfarin, or placebo in venous thromboembolism. N Engl J Med. 2013;368(8):709–718. 23425163 10.1056/NEJMoa1113697

[52] Available from: https://clinicaltrials.gov/ct2/show/NCT00329238?term=NCT00329238&draw=2&rank=1

[53] Weitz JI, Lensing AWA, Prins MH, et al. Rivaroxaban or aspirin for extended treatment of venous thromboembolism. N Engl J Med. 2017;376(13):1211–1222. 28316279 10.1056/NEJMoa1700518

