# Supplementary material for: Antithrombotic therapy for secondary prevention of unprovoked venous thromboembolism: a systematic review and network meta-analysis of randomized controlled trials
Source: Ann Med. 2022 Jan 13;54(1):253–61. doi: 10.1080/07853890.2022.2026002 (PMC8759723; doi:10.1080/07853890.2022.2026002)

**Antithrombotic therapy for secondary prevention of unprovoked venous thromboembolism: a systematic review and network meta-analysis of randomized controlled trials**

**Supplemental data**

**Table S1. Search strategies of systematic review**

**Table S2. Ranking of different antithrombotic strategies based on surface under the cumulative ranking curves and mean ranks.**

**Table S3. Global inconsistency among comparisons for efficacy and safety of different antithrombotic strategies for unprovoked venous thromboembolism.**

**Table S4. Meta regression of possible risk factors on the efficacy and safety of different antithrombotic treatments on unprovoked venous thromboembolism. Data were given as P values.**

**Table S5. Pairwise meta-analysis results of efficacy and safety of different antithrombotic strategies for unprovoked venous thromboembolism. OR: odds ratio; CI: confidence interval.**

**Table S6. Network meta-analysis results of recurrent venous thromboembolism and all-cause death. Data were given as odds ratio and 95% confidence intervals.**

**Figure S1. Risk of bias summary: review authors' judgments about each risk of bias item for each included study**

**Figure S2. Network of eligible comparisons for efficacy and safety of different antithrombotic strategies for unprovoked venous thromboembolism.**

**Figure S3. Sensitivity analysis of studies involving 100% unprovoked venous thrombosis patients. VTE: venous thromboembolism; OR: odds ratio; CI: confidence interval**

**Table S1. Search strategies of systematic review**

| **Search strategies using the OVID interface** |
| --- |
| 1. Venous Thrombosis/  2. Thrombosis/  3. Thromb$.ab.  4. phlebothrombosis.ab.  5. Deep vein thrombosis.ab.  6. Pulmonary Embolism/  7. (PE or DVT or VTE).ab.  8. or/1-7  9. Anticoagulant/  10. (anticoagul$ or anti-coagu$ or antithrombotic$).ab.  11. (warfarin or VKA).ab.  12. (dabigatran or Pradaxa or Pradax or rivaroxaban or Xarelto or Apixaban or Eliquis or Savaysa or edoxaban).ab.  13. or/9-12  14. (extend$ or prolong$).ab.  15. duration.ab.  16. long$.ab.  17. continue.ab.  18. indefinite.ab.  19. or/14-18  20. 8 and 13 and 19  21. limit 20 to (english language and randomized controlled trial) |
| **Search strategies of the Cochrane Library** |
| 1. “Venous Thrombosis”  2. “Thrombosis”  3. “Thromb$”:ab  4. “phlebothrombosis”:ab  5. “Deep vein thrombosis”:ab  6. “Pulmonary Embolism”  7. (PE or DVT or VTE):ab  8. #1 or #2 or #3 or #4 or #5 or #6 or #7  9. “Anticoagulant”  10. (anticoagul$ or anti-coagu$ or antithrombotic$):ab  11. (warfarin or VKA):ab  12. (dabigatran or Pradaxa or Pradax or rivaroxaban or Xarelto or Apixaban or Eliquis or Savaysa or edoxaban):ab  13. #9 or #10 or #11 or #12  14. (extend$ or prolong$):ab  15. duration:ab  16. long$:ab  17. continue:ab  18. indefinite:ab  19. #14 or #15 or #16 or #17 or #18  20. #8 and #13 and #19  With Content type of “in trials” |

**Table S2. Ranking of different antithrombotic strategies based on surface under the cumulative ranking curves and mean ranks.**

| Treatments | Major bleeding | | | Recurrent VTE | | | All-cause death | | |
| --- | --- | --- | --- | --- | --- | --- | --- | --- | --- |
|  | SUCRA | PrBest | MeanRank | SUCRA | PrBest | MeanRank | SUCRA | PrBest | MeanRank |
| Placebo/observation | 67.4 | 2.3 | 3.3 | 5.5 | 0 | 7.6 | 24.4 | 0 | 6.3 |
| Apixaban | 90.6 | 66.4 | 1.7 | 68.8 | 21.2 | 3.2 | 85.7 | 44.4 | 2 |
| Low-apixaban | 81.3 | 25.7 | 2.3 | 70 | 23.2 | 3.1 | 66.9 | 8.4 | 3.3 |
| Rivaroxaban | 12.3 | 1.3 | 7.1 | 69 | 26.8 | 3.2 | 59.7 | 30.9 | 3.8 |
| Warfarin | 22.7 | 0 | 6.4 | 57.7 | 2.9 | 4 | 37.2 | 0.4 | 5.4 |
| Low-warfarin | 32.3 | 1.4 | 5.7 | 46.4 | 8.5 | 4.8 | 65 | 15.1 | 3.5 |
| Aspirin | 59 | 2.8 | 3.9 | 21.4 | 0.2 | 6.5 | 29.9 | 0.3 | 5.9 |
| Edoxaban | 34.4 | 0.1 | 5.6 | 61.3 | 17.2 | 3.7 | 31.1 | 0.5 | 5.8 |

SUCRA: Surface under the cumulative ranking curves; VTE: venous thromboembolism

**Table S3. Global inconsistency among comparisons for efficacy and safety of different antithrombotic strategies for unprovoked venous thromboembolism.**

| **Items** | **P value** |
| --- | --- |
| Major bleeding | 0.566 |
| Recurrent VTE | 0.851 |
| All-cause death | 0.128 |

**Table S4. Meta regression of possible risk factors on the efficacy and safety of different antithrombotic strategies for unprovoked venous thromboembolism. Data were given as P values.**

| **Risk factors** | **Major bleeding** | **Recurrent VTE** | **All-cause death** |
| --- | --- | --- | --- |
| Race | 0.818 | 0.35 | NA |
| Age | 0.58 | 0.469 | 0.456 |
| Gender | 0.51 | 0.776 | 0.372 |
| Episodes | 0.373 | 0.828 | 0.712 |
| Percentage of unprovoked VTE | 0.948 | 0.977 | 0.588 |
| VTE categories | 0.667 | 0.704 | 0.816 |
| Treatment duration | 0.227 | 0.644 | 0.847 |

VTE: venous thromboembolism

**Table S5. Pairwise meta-analysis results of efficacy and safety of different strategies for unprovoked venous thromboembolism. OR: odds ratio; CI: confidence interval.**

| Comparisons | Major bleeding | | | | Recurrent VTE | | | | All-cause death | | | |
| --- | --- | --- | --- | --- | --- | --- | --- | --- | --- | --- | --- | --- |
|  | N | OR (95% CI) | I^2^ | P | N | OR (95% CI) | I^2^ | P | N | OR (95% CI) | I^2^ | P |
| Warfarin vs Placebo/observation | 10 | 2.79 (1.34, 5.80)^*^ | 0.0 | 0.986 | 10 | 0.24 (0.12, 0.49)^*^ | 66.6 | 0.001 | 6 | 0.88 (0.40, 1.94) | 0.0 | 0.759 |
| Apixaban vs Placebo/observation | 1 | 0.25 (0.03, 2.28) | - | - | 1 | 0.18 (0.10, 0.32)^*^ | - | - | 1 | 0.29 (0.09, 0.88)^*^ | - | - |
| Low-apixaban vs Placebo/observation | 1 | 0.49 (0.09, 2.69) | - | - | 1 | 0.18 (0.10, 0.31)^*^ | - | - | 1 | 0.49 (0.20, 1.22) | - | - |
| Low-apixaban vs apixaban | 1 | 1.94 (0.18, 21.41) | - | - | 1 | 0.97 (0.46, 2.04) | - | - | 1 | 1.70 (0.50, 5.83) | - | - |
| Rivaroxaban vs Placebo/observation | 1 | 8.94 (0.48, 166.41) | - | - | 1 | 0.18 (0.08, 0.38)^*^ | - | - | 1 | 0.49 (0.04, 5.45) | - | - |
| Aspirin vs Placebo/observation | 2 | 1.19 (0.51, 2.77) | 0.0 | 0.711 | 2 | 0.68 (0.50, 0.92)^*^ | 0 | 0.404 | 2 | 0.95 (0.52, 1.72) | 0.0 | 0.703 |
| Edoxaban vs Warfarin | 1 | 0.85 (0.59, 1.21) | - | - | 1 | 0.89 (0.70, 1.13) | - | - | 1 | 1.05 (0.82, 1.35) | - | - |
| Low-warfarin vs Warfarin | 1 | 1.13 (0.43, 2.96) | - | - | 1 | 2.74 (1.06, 7.09)^*^ | - | - | 1 | 2.05 (0.86, 4.84) | - | - |
| Low-warfarin vs Placebo/observation | 1 | 2.51 (0.48, 13.06) | - | - | 1 | 0.34 (0.18, 0.64)^*^ | - | - | 1 | 0.49 (0.15, 1.64) | - | - |

^*^Results with significant differences. VTE: venous thromboembolism

**Table S6. Network meta-analysis results of recurrent venous thromboembolism and all-cause death. Data were given as odds ratio and 95% confidence intervals.**

1. **Recurrent VTE during treatment**

| Edoxaban |  |  |  |  |  |  |  |
| --- | --- | --- | --- | --- | --- | --- | --- |
| 0.34 (0.05,2.32) | Aspirin |  |  |  |  |  |  |
| 0.66 (0.07,6.14) | 1.92 (0.29,12.77) | Low-warfarin |  |  |  |  |  |
| 0.89 (0.21,3.79) | 2.60 (0.75,9.08) | 1.35 (0.25,7.41) | Warfarin |  |  |  |  |
| 1.26 (0.13,12.23) | 3.69 (0.53,25.60) | 1.92 (0.20,18.28) | 1.42 (0.25,8.17) | Rivaroxaban |  |  |  |
| 1.27 (0.14,11.67) | 3.72 (0.57,24.19) | 1.93 (0.21,17.44) | 1.43 (0.27,7.66) | 1.01 (0.11,9.46) | Low-apixaban |  |  |
| 1.23 (0.13,11.29) | 3.60 (0.55,23.40) | 1.87 (0.21,16.87) | 1.38 (0.26,7.41) | 0.98 (0.10,9.15) | 0.97 (0.19,4.86) | Apixaban |  |
| 0.65 (0.23,1.89) | 0.34 (0.07,1.63) | 2.03 (0.18,22.75) | 0.25 (0.13,0.49)^*^ | 0.18 (0.03,0.90)^*^ | 0.18 (0.04,0.82)^*^ | 0.18 (0.04,0.85)^*^ | Placebo/observation |

^*^Results with significant differences.

1. **All-cause death during treatment**

| Edoxaban |  |  |  |  |  |  |  |
| --- | --- | --- | --- | --- | --- | --- | --- |
| 0.98 (0.36,2.70) | Aspirin |  |  |  |  |  |  |
| 1.89 (0.44,8.19) | 1.02 (0.37,2.82) | Low-warfarin |  |  |  |  |  |
| 1.05 (0.82,1.35) | 1.07 (0.40,2.87) | 0.55 (0.13,2.35) | Warfarin |  |  |  |  |
| 1.88 (0.15,23.78) | 1.92 (0.16,22.83) | 0.99 (0.07,14.63) | 1.79 (0.14,22.37) | Rivaroxaban |  |  |  |
| 1.89 (0.55,6.44) | 1.93 (0.65,5.75) | 1.00 (0.22,4.55) | 1.80 (0.54,5.98) | 1.01 (0.08,13.16) | Low-apixaban |  |  |
| 3.21 (0.81,12.82) | 3.28 (0.93,11.64) | 1.70 (0.33,8.81) | 3.06 (0.78,11.94) | 1.71 (0.12,24.20) | 1.70 (0.50,5.83) | Apixaban |  |
| 0.95 (0.52,1.72) | 0.49 (0.15,1.64) | 3.69 (0.69,19.80) | 0.88 (0.40,1.92) | 0.49 (0.04,5.45) | 0.49 (0.20,1.22) | 0.29 (0.09,0.88)^*^ | Placebo/observation |

^*^Results with significant differences.

**Figure S1.**


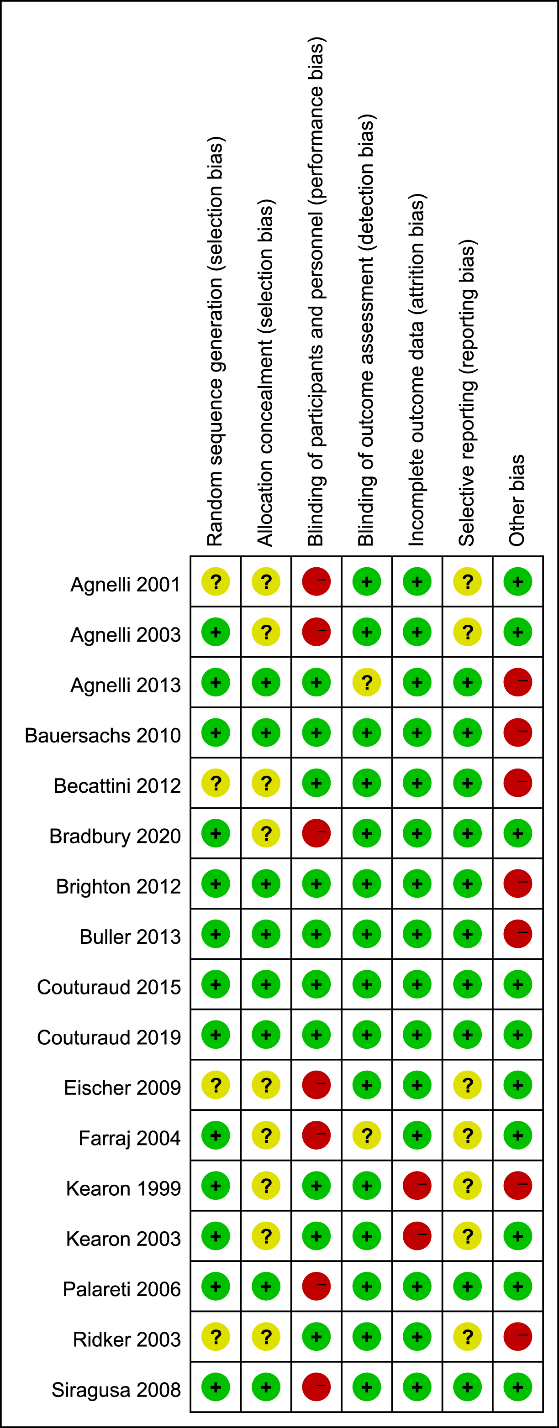


Figure S2.


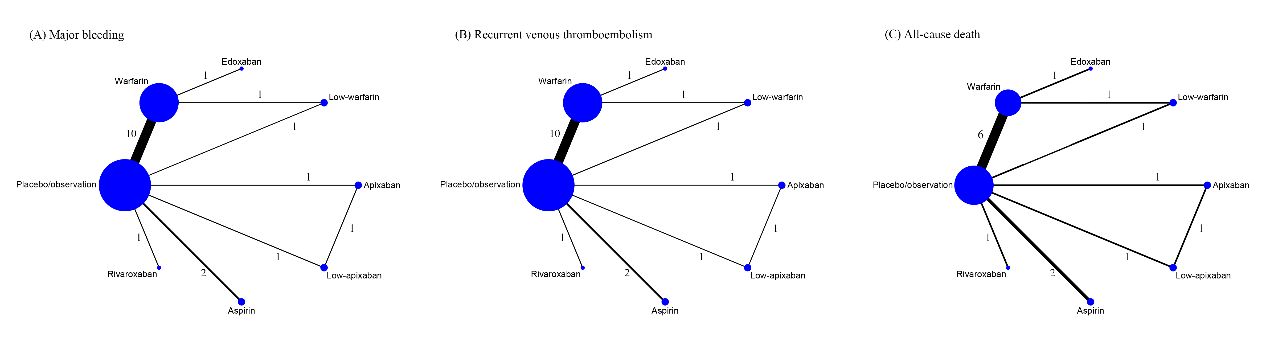


**Figure S3.**


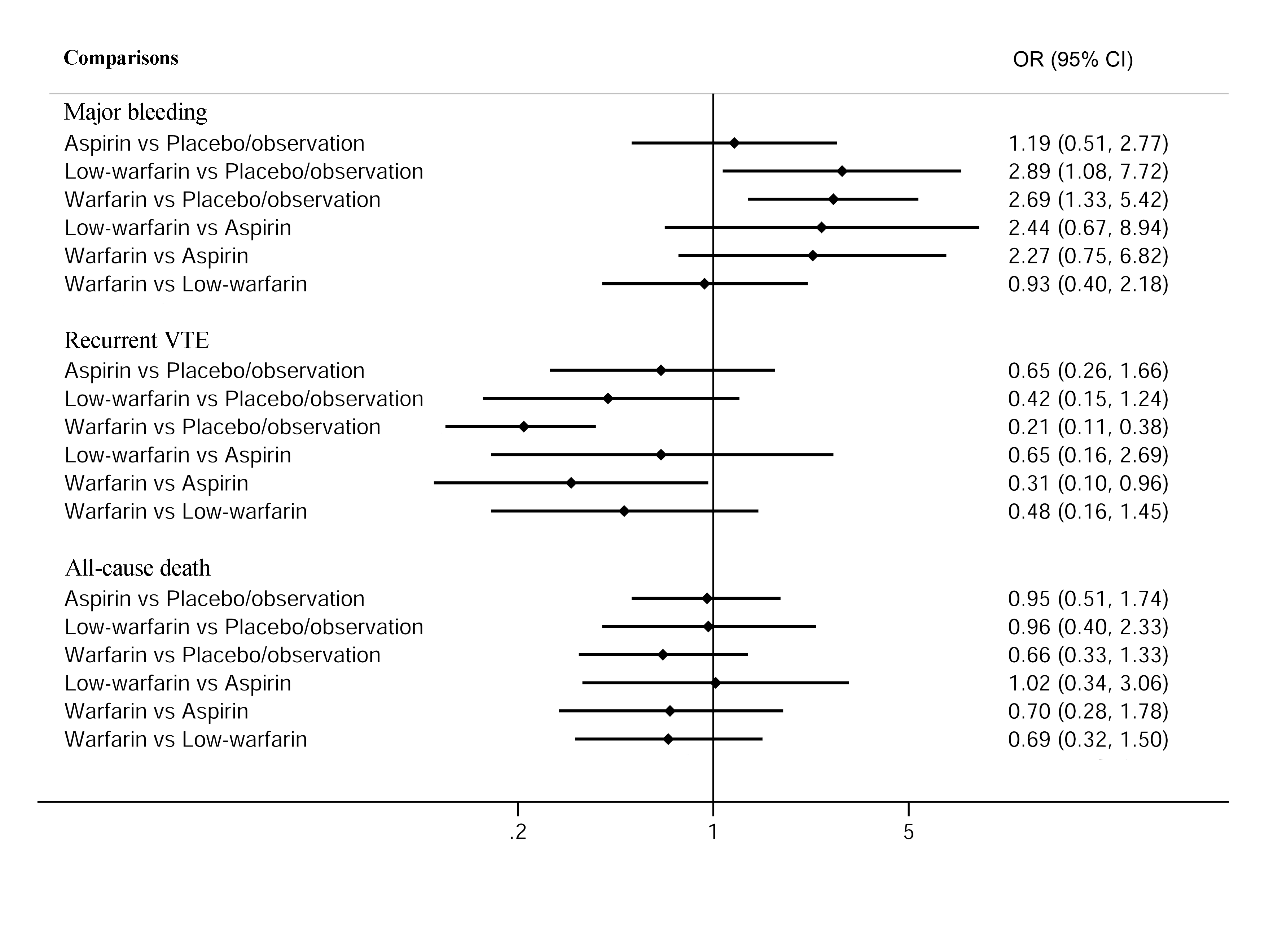

Supplement: Supplemental Material [file IANN_A_2026002_SM6491.docx]
